# No causal association between gut microbiota and kawasaki disease: a two-sample mendelian randomization study

**DOI:** 10.1038/s41390-025-03878-5

**Published:** 2025-01-25

**Authors:** Sibao Wang, Gang Luo, Zhixian Ji, Silin Pan

**Affiliations:** Heart Center, Women and Children’s Hospital, Qingdao University, Qingdao, China

## Abstract

**Background:**

Despite prior observational studies suggesting a link between gut microbiota to Kawasaki disease (KD), these findings remain debated. This study aimed to assess the association between gut microbiota and KD on a genetic level using a two-sample Mendelian randomization (MR) analysis.

**Methods:**

This two-sample MR analysis utilized summary statistics from the largest genome-wide association study meta-analysis on gut microbiota conducted by the MiBioGen consortium. The causal relationship between gut microbiota and KD evaluated using multiple methods, including inverse variance weighted (IVW), MR Egger, weighted median, simple mode, weighted mode, and MR-PRESSO. Reverse MR analysis was conducted on bacteria identified as causally linked to KD in the initial MR study. Cochran’s Q and Rucker’s Q tests assessed the heterogeneity among instrumental variables.

**Results:**

The IVW estimates indicated no significant genetic causal relationship with KD for various taxa, including genus *Bifidobacterium* (*p* = 0.774, OR 95% CI = 0.876 [0.355–2.163]), genus *FamilyXIIIAD3011group* (*p* = 0.945, OR 95% CI = 0.979 [0.539–1.780]), genus *LachnospiraceaeUCG004* (*p* = 0.987, OR 95%CI = 1.005 [0.542–1.863]), genus RuminococcaceaeNK4A214group (*p* = 0.453, OR 95%CI = 1.469 [0.538–4.009]), genus *RuminococcaceaeUCG002* (*p* = 0.835, OR 95% CI = 1.092 [0.478–2.494]), genus *LachnospiraceaeUCG001* (*p* = 0.996, OR 95%CI = 0.998 [0.482–2.066]), genus *Bacteroides* (*p* = 0.595, OR 95%CI = 0.831 [0.419–1.648]), genus Olsenella (*p* = 0.414, OR 95%CI = 1.312 [0.684–2.516]), genus *Lactococcus* (*p* = 0.870, OR 95%CI = 0.962 [0.600–1.541]), family *Rhodospirillaceae* (*p* = 0.995, OR 95%CI = 1.002 [0.550–1.827]), family *FamilyXIII* (*p* = 0.894, OR 95%CI = 1.093 [0.298–4.009]), family *BacteroidalesS24* (*p* = 0.604, OR 95%CI = 0.849 [0.456–1.578]), family *Ruminococcaceae* (*p* = 0.524, OR 95%CI = 0.692 [0.223–2.148]), and class *Bacilli* (*p* = 0.905, OR 95%CI = 0.967 [0.561–1.667]). The reverse MR analysis revealed no significant causal effect of KD on gut microbiota. No significant heterogeneity of instrumental variables or horizontal pleiotropy was observed.

**Conclusion:**

This bidirectional causal inference analysis did not reveal a genetic causal relationship between gut microbiota and KD. Confounding factors may have influenced the observed associations in previous observational studies. Further research with advanced MR methods and larger GWAS datasets is needed to confirm these findings.

**Impact:**

- This study employs Mendelian randomization to investigate the causal relationship between gut microbiota and Kawasaki disease, and finds no evidence of a genetic association between them.
- This represents the first Mendelian randomization study to examine the causal link between gut microbiota and Kawasaki disease, offering valuable insights into the potential mechanisms underlying previous observational findings.
- The study challenges existing observational findings by suggesting that the association between gut microbiota and Kawasaki disease may be confounded by other factors, thereby highlighting the necessity for further genetic studies to elucidate the role of gut microbiota in the disease.

## Introduction

Kawasaki disease (KD) is an acute febrile illness that primarily affecting children. First described in 1967 by Dr. Tomisaku Kawasaki,^1^ KD manifests globally across all racial groups. Notably, developed nations have reported an increasing incidence of KD, which is now the leading cause of childhood-acquired heart disease in these regions.^2^ KD is a multisystemic disease that primarily affects the coronary arteries, potentially leading to stenosis, coronary artery aneurysm, and thrombosis.^3^ Epidemiological data reveal that untreated children exhibit a KD incidence rate of 15–20%. However, this rate drops to 4% with effective treatment combining intravenous gamma globulin and aspirin.^2^ Despite extensive research, the etiology of KD remains elusive. Studies suggest it is an immune-mediated condition often triggered post-infection in genetically susceptible individuals. Recent research has associated the pathogenesis of the disease with inflammatory cytokines, genetic variations, and immune system activation.^4^ A growing body of evidence suggests that genetic predispositions and microbial infections may collectively influence the development of KD.

The gastrointestinal tract, a vital metabolic organ, houses a diverse array of microorganisms essential for nutrient processing, immune development, colonization resistance, and various other physiological functions critical to maintaining health and homeostasis.^5^ In children, the developing gut microbiota plays a crucial role in immune development, and disruptions can make children more susceptible to infections and diseases like KD.^6^ Numerous studies have reported gut microbiota dysbiosis in patients with KD.^7^ However, these studies are limited by their cross-sectional design, which hinders the ability to determine whether the observed dysbiosis is a result of KD or a preexisting condition of the intestinal microbiota before the onset of KD. This limitation primarily stems from the inherent challenges posed by confounding factors and reverse causality bias in such studies. Establishing a causal link between gut microbiota and KD could offer significant insights and potential breakthroughs in disease prevention, diagnosis, and treatment.

Mendelian Randomization (MR) presents a robust analytical approach to overcome these challenges. MR leverages genetic variants as instrumental variables (IVs) by inferring causal relationships between potentially modifiable exposures and outcomes. MR allows causal inference to be made based on genetic variants, which are not influenced by confounders or reverse causality. This method effectively mitigates confounding factors and addresses reverse causality issues, which have been persistent obstacles in observational research.^8^ By employing MR, this study aims to provide more definitive evidence of the causal relationship between gut microbiota and KD. Specifically, we will conduct a bidirectional two-sample MR analysis using publicly available summary statistics from large-scale genome-wide association studies (GWAS). This innovative approach will allow us to genetically assess the exact association between gut microbiota and KD, thereby contributing valuable insights into the pathogenesis of KD, and potentially guiding future therapeutic strategies.

## Methods

### Study design

MR utilizes genetic variants, primarily single-nucleotide polymorphisms (SNPs), as IVs to investigate the genetic association between exposure and outcome. This approach relies on three fundamental assumptions: (1) genetic variants are highly correlated with the exposure, (2) genetic variants are independent of potential confounders, (3) genetic variants solely affect outcomes through exposure. IVs are deemed valid only when these assumptions are met. This study employed a bidirectional two-sample MR analysis to evaluate the genetic association between gut microbiota and KD. Initially, SNPs associated with gut microbiota were used to examine their effects on KD. Subsequently, to explore the possibility of reverse association, appropriate IVs were used to quantify the implications of KD on gut microbiota. All datasets used in this study are publicly available.

### Exposure GWAS datasets

The MiBioGen consortium, which conducted the most extensive genome-wide meta-analysis of gut microbiota composition, contributed to the genetic variations of gut microbiota in this study. This meta-analysis probed the relationship between human autosomal genetic variations and the gut microbiome, by profiling the microbial composition of 18,340 participants across 24 independent cohorts, with most participants belonging to a European ancestry (*n* = 13,266).^9^ A comprehensive set of 211 taxa (9 phyla, 16 classes, 20 orders, 35 families, and 131 genera) were incorporated into this meta-analysis. The data are publicly accessible via the website (www.mibiogen.org).

### Outcome GWAS datasets

Genetic associations for KD were obtained from the largest available GWAS meta-analysis conducted by Hoggart and colleagues,^10^ comprising 400 cases and 6101 controls. The team went ahead to build a new KD susceptibility GWAS, by amalgamating the recently genotyped KD cases and comparing them with healthy controls. Genome-wide summary statistics for the KD susceptibility meta-analysis are available for downloaded from the EBI GWAS Catalog (https://www.ebi.ac.uk/gwas/studies/GCST90013537).

### Instrumental variables

We executed a series of rigorous quality controls to identify IVs that meet the three assumptions of MR analysis, hence ensuring the robustness and reliability of said analysis. First, SNPs with a significant association with the gut microbiome were designated as IVs. Since only a minor fraction of the gut microbiome exhibits more than three independent SNPs at a genome-wide significant threshold of a *p* < 5 × 10^−8^, we made decided to increase the *p*-value threshold to 1 × 10^−5^ to obtain the required number of IVs and generate more comprehensive results.^11,12^ Secondly, SNPs with a minor allele frequency (MAF) of less than 0.01 were excluded. Thirdly, to circumvent IVs linked with linkage disequilibrium (LD), SNPs linked to LD were assessed using the European 1000 Genomes Project reference panel, with the requirements of *r*^2^ < 0.001 and a clump spacing greater than 10,000 kb.^13^ Fourthly, to assert a powerful association with exposure, we picked SNPs showcasing an *F* statistic >10 as IVs. *F* statistics were computed using the formula F = *R*^2^(n−k−1)/k(1−*R*^2^), where *R*^2^ symbolizes the proportion of variance explained by instruments, n stands for the sample size, and k denotes the count of selected IVs.^14^ Phenoscanner is utilized to identify potential confounding factors.^15^

### Statistical analysis

Using the selected IVs, two-sample MR analyzes were conducted on gut microbiota and KD using the TwoSampleMR and MR-PRESSO packages in R (version 4.3.1). The MR analysis was performed using five distinct methods: the primary method employed was random-effects inverse variance weighted (IVW), while auxiliary methods included MR Egger, weighted median, simple mode, and weighted mode. The results from the random-effects IVW served as the foundation of our study. To evaluate the heterogeneity of our MR analysis, we calculated Cochran’s Q statistic for the IVW method and Rucker’s Q statistic for the MR Egger method. A *p*-value > 0.05 indicated no significant heterogeneity.^16^ We utilized the intercept test from MR Egger to assess horizontal pleiotropy, with a *p*-value exceeding 0.05 suggesting the absence of horizontal pleiotropy. In addition to these tests, the MR pleiotropy residual sum and outlier (MR-PRESSO) method was employed, which is capable of detecting both horizontal pleiotropy and outliers within our data.^17^ Leave-one-out analysis was conducted to examine sensitivity to individual SNPs.^18^ To evaluate the causative link between gut microbiota and KD, we also enacted a reverse MR analysis on the bacteria found to be causally related with KD in forward MR analysis. The global test of MR-PRESSO analysis was utilized to conduct the horizontal pleiotropy test, and a *p*-value > 0.05 denoted no horizontal pleiotropy, and the distortion test of MR-PRESSO analysis was deployed to determine whether outliers existed in our MR analysis.^19^ Overall, our study’s findings are primarily based on the random-effects IVW results, with rigorous consideration of potential biases due to horizontal pleiotropy and outliers.

## Results

### IVs Selection

A series of filtering steps were applied, including exclusions based on MAF < 0.01, quality control metrics (e.g., imputation quality), and removal of SNPs in LD (*r*² < 0.001). After these steps, a total of 116 SNPs across various taxonomic levels, including class, family, and genus, were selected. Following clumping and harmonization processes, the count of SNPs related to spinal stenosis ranged between 6 to 11. Moreover, the family *BacteroidalesS24* has the greatest number of SNPs (*n* = 11), whereas the genus *LachnospiraceaeUCG001*, family *FamilyXIII*, and family *Ruminococcaceae* have the smallest number of SNPs (*n* = 6). The MAFs of all the aforementioned SNPs exceeded 0.01. Detailed characteristics of each SNP, including effect allele, other allele, beta, standard error, p-value, and F-statistic, are provided in Supplementary Table 1. SNP analysis in PhenoScanner indicated no association with confounders (Supplementary Table 2).

### MR Analysis

**Fig. 1:**
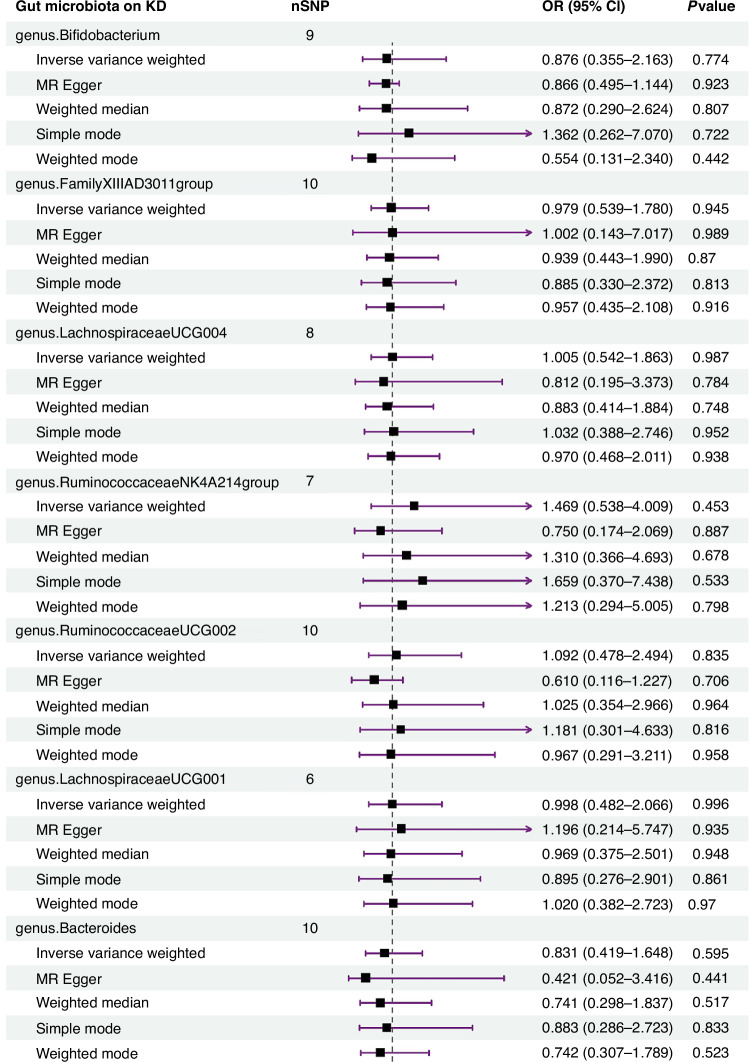

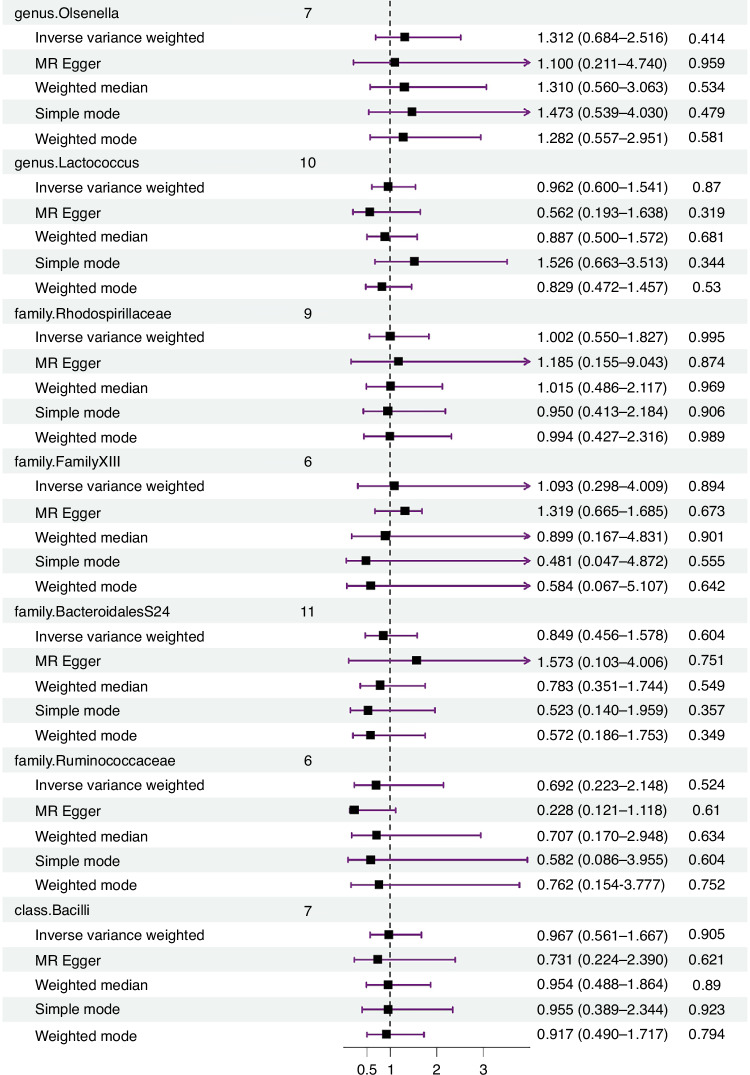
Forest plot of MR analysis between the gut microbiota and KD. Random effects IVW, MR Egger, weighted median, simple model, and weighted model analyzes showed no genetic causal correlation between gut microbiota and KD (*p* > 0.05).

The random-effects IVW analysis found no significant genetic causal associations between KD and various taxa, including genus *Bifidobacterium* (*p* = 0.774, OR 95% CI = 0.876 [0.355–2.163]), genus *FamilyXIIIAD3011group* (*p* = 0.945, OR 95% CI = 0.979 [0.539–1.780]), genus *LachnospiraceaeUCG004* (*p* = 0.987, OR 95%CI = 1.005 [0.542–1.863]), genus *RuminococcaceaeNK4A214group* (*p* = 0.453, OR 95%CI = 1.469 [0.538–4.009]), genus *RuminococcaceaeUCG002* (*p* = 0.835, OR 95% CI = 1.092 [0.478–2.494]), genus *LachnospiraceaeUCG001* (*p* = 0.996, OR 95%CI = 0.998 [0.482–2.066]), genus *Bacteroides* (*p* = 0.595, OR 95%CI = 0.831 [0.419–1.648]), genus *Olsenella* (*p* = 0.414, OR 95%CI = 1.312 [0.684–2.516]), genus *Lactococcus* (*p* = 0.870, OR 95%CI = 0.962 [0.600–1.541]), family *Rhodospirillaceae* (*p* = 0.995, OR 95%CI = 1.002 [0.550–1.827]), family *FamilyXIII* (*p* = 0.894, OR 95%CI = 1.093 [0.298–4.009]), family *BacteroidalesS24* (*p* = 0.604, OR 95%CI = 0.849 [0.456–1.578]), family *Ruminococcaceae* (*p* = 0.524, OR 95%CI = 0.692 [0.223–2.148]), and class *Bacilli* (*p* = 0.905, OR 95%CI = 0.967 [0.561–1.667]) manifested no genetic causal correlation with KD. Analysis leveraging MR Egger, weighted median, simple mode, and weighted mode corroborated the random-effects IVW findings (Fig. 1 and Supplementary Table 3).

**Fig. 2:**
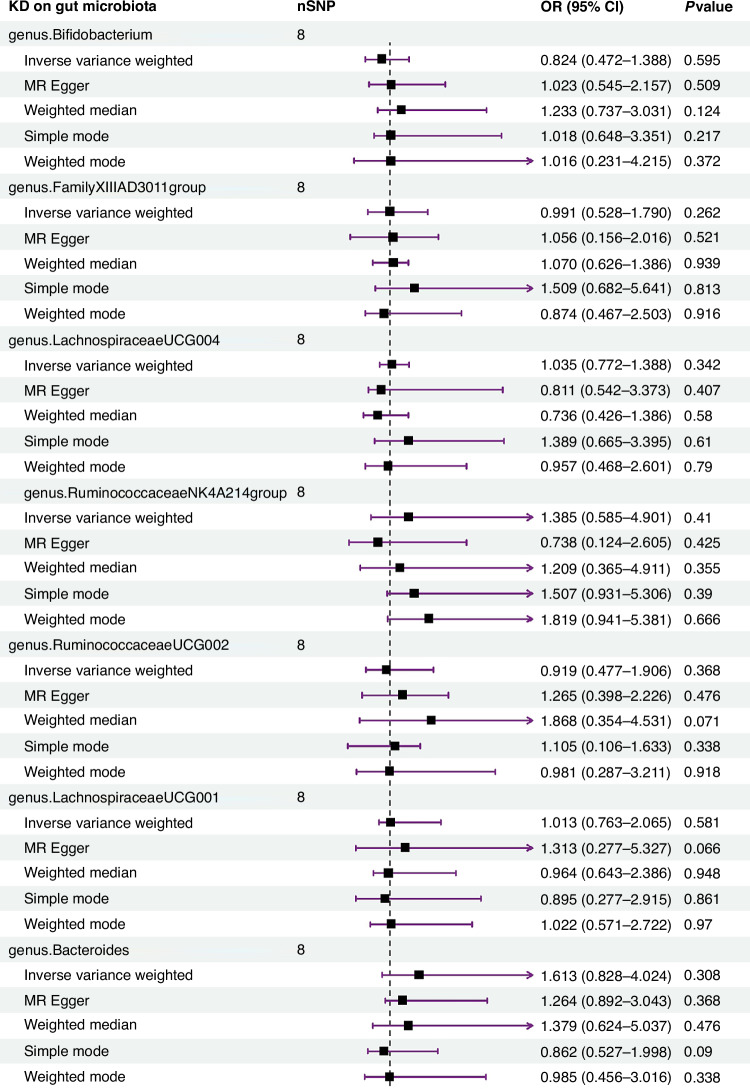

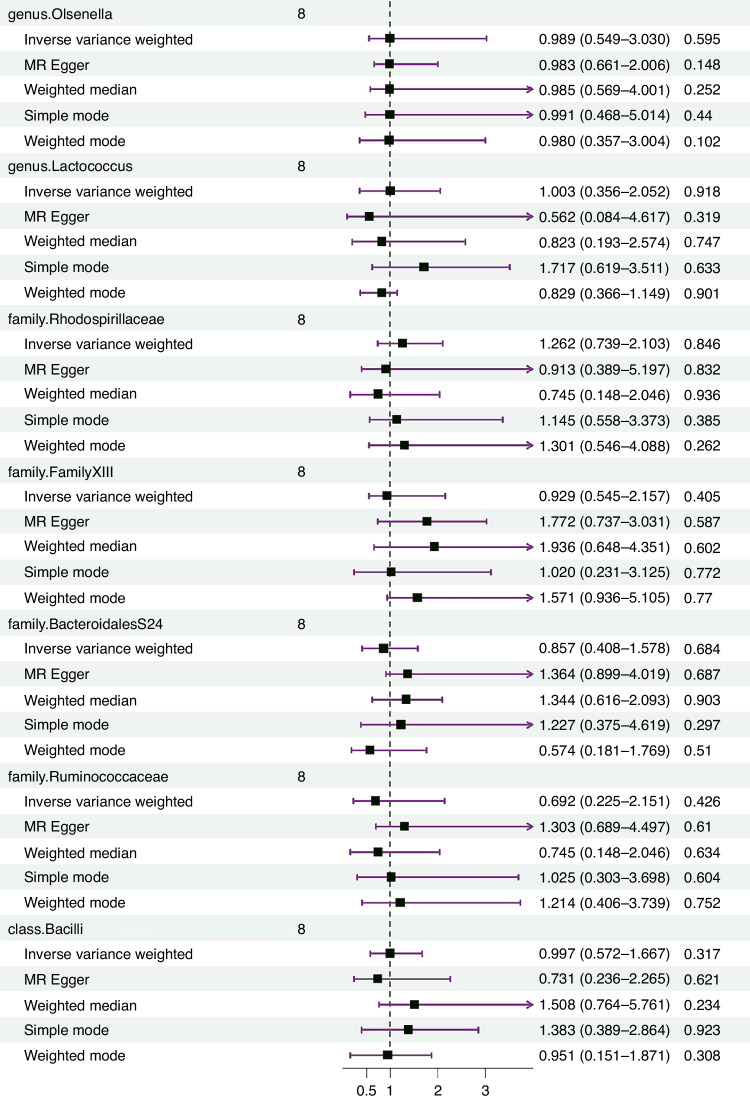
Forest plot of reverse MR analysis between the gut microbiota and KD. Random effects IVW, MR Egger, weighted median, simple model, and weighted model analyzes showed no genetic causal correlation between KD and gut microbiota (*p* > 0.05).

The reverse MR analysis indicated no significant causal relationship between KD and the identified gut microbiota taxa. The outcomes hinted at promising non-significance (*p* > 0.05) for heterogeneity as per Cochran’s IVW Q-test, and suggest directional horizontal pleiotropy under the MR-Egger regression intercept. Moreover, comprehensive details about the chosen IVs and MR findings concerning reverse MR analysis are presented in Fig. 2 and Supplementary Table 4.

### Pleiotropy and sensitivity analysis

Both Cochran’s Q statistic (MR-IVW) and Rucker’s Q statistic affirmed an absence of heterogeneity in the MR analyzes of the IVs and outcomes (*p* > 0.05)(Supplementary Table S3,S4). The MR-Egger regression, together with the MR-PRESSO global test, indicated a lack of horizontal pleiotropy between the IVs and outcomes (*p* > 0.05)(Supplementary Table S3,S4). The leave-one-out analysis suggested that the results were not driven by any single SNPs. The results of the pleiotropic and sensitivity analyzes are presented in Supplementary Figs. S1–S3.

## Discussion

In this study, we conducted a two-sample MR analysis using summary statistics of gut microbiota from the largest GWAS meta-analysis by the MiBioGen consortium and summary statistics of KD from the NHGRI-EBI Catalog of human genome-wide association studies. This approach allowed us to assess the causal relationship between gut microbiota and KD from a genetic perspective, addressing the limitations of traditional observational studies. Utilizing expansive GWAS datasets, our MR inquiry aims to bridge this scholarly void from an innovative vantage point. To our knowledge, this is the first MR study to investigate the potential causal relationship between gut microbiota and KD. Interestingly, our results did not find evidence supporting a causal association between gut microbiota and KD in individuals of European descent, constituting a significant contribution to the genetic research of KD.

There is no agreement on whether KD-related infectious agents are of viral, bacterial, or fungal origin,^20^ and the underlying immune mechanisms behind KD have not been completely elucidated, remaining only partially understood. The absence of a proven unambiguous cause of KD has prompted the scientific community to explore other hypothetical environmental triggers. Particularly, the composition of the resident intestinal flora as a potential contributor to KD has been evaluated by different research groups. Although gut microbiota has been reported to be associated with KD pathogenesis, these speculations have been controversial.^21^ It has been suggested that colonization by normal microbiota variants could induce dysregulation in the immune systems of children with a pre-existing genetic defect in immune maturation, leading to a hyperimmune reaction and the development of KD.^22^ While these studies have highlighted potential links, they are inherently susceptible to confounding factors and biases that may distort causal interpretations. However, it is important to note that our analysis does not rule out the possibility that gut microbiota may play a role in KD; rather, it emphasizes that the evidence for a direct genetic causal relationship is currently lacking.

Given that the gastrointestinal tract serves as the primary interface between microbial elements and their host, housing the majority of bacteria and the most significant quantity of lymphoid tissue in the body, Eladawy et al. proposed a hypothesis that the intestinal environment could undergo modifications in children suffering from KD, and it was indeed observed that KD patients exhibited a higher prevalence of gastrointestinal symptoms and complications.^23^ More specifically, Takeshita et al. ^24^ conducted an evaluation on 20 patients with KD, 20 patients exhibiting acute febrile diseases, and 20 healthy children. They discovered the incidence of Lactobacilli isolated from KD patients (2/20, 10%) was significantly lower (*p* < 0.001) than in the other cohorts. Additionally, the presence of Staphylococcus, Streptococcus, Enterococcus, Enterobacteriaceae, Bifidobacterium were found among the three groups. In contrast, the detection rate of Eubacterium and Peptostreptococcus was noticeably elevated in KD patients compared with patients suffering from other febrile diseases (*p* < 0.01 and *p* < 0.05, respectively). However, no significant contrasts were identified between KD patients and healthy children.^24^

However, the results of our bidirectional causal inference analysis showed no support for a genetically predicted causal relationship between gut microbiota and KD. While this differs from our previous findings, we believe this outcome to be valid. We propose three main factors contributing to this outcome. First, the microbiota data used in our study may lack the resolution necessary to detect associations with KD, given that only a limited number of SNPs were available. Second, their association observed in previous observational studies may have been caused by confounding factors. Third, the interplay of genetic predisposition and environmental factors in the pathogenesis of KD parallels the complexities seen in other multifactorial diseases. The primary obstacle in today’s medical research is determining whether microbiota modifications during KD cause the disease or are merely a marker and consequence of it. Thus, more advanced MR analysis methods, larger-scale GWAS summary data, and more genetic instruments are needed to validate the findings of this study.

KD is recognized as a multifactorial disease, likely developing in genetically or environmentally susceptible individuals following an exaggerated immune response to infection.^25^ Cesarean delivery,^26^ formula feeding,^27^ and a history of antimicrobial use prior to KD onset^26^ constitute reported environmental risk factors contributing to the disease’s development. These factors also significantly influence the intestinal microbiota, thus leading to the hypothesis that disturbances in the gut microbiota, i.e., dysbiosis, might increase susceptibility to KD. There have been two previous reports on the dysbiosis in patients with KD.^7,28^ However, all studies have encompassed patients in the acute phase of KD. One study identified transient changes in intestinal microbiota during the rise in body temperature, which is common with viral infections.^29,30^ As a result, determining whether the dysbiosis in the acute phase of KD is a consequence of KD or a precipitating factor remains a challenge.

To clarify the causal interpretation of our findings, it is essential to understand the potential limitations of observational studies. Observational studies, while useful in generating hypotheses, may be prone to confounding biases and reverse causality, leading to less robust conclusions. In contrast, MR analysis offers a more rigorous approach by leveraging genetic variants as instrumental variables to infer causality, thus providing a more reliable estimate despite its current limitations. However, it is important to note that our study was confined to populations in Europe and did not consider age distinctions, which are crucial when interpreting results related to pediatric populations. Additionally, the relatively small sample size of gut microbiota data could have affected the precision of the reverse MR analysis results. While our study focused on genetic factors associated with gut microbiota, we recognize that the bacterial structure and metabolites may also play important roles in KD pathogenesis. Future studies integrating microbiome profiling and metabolomic analyzes may provide further insights into the mechanisms underlying these relationships.

Our study has several limitations. Initially, our research was confined to populations in Europe; thus, when extending our conclusions to diverse populations, careful consideration is imperative. Age distinction was absent in our study, and it’s crucial when interpreting our results, especially when relating them to the pediatric population solely. Our extant research couldn’t segregate intestinal flora depending on age. Further, the gut microbiota’s sample size was rather small, potentially interfering with the precision of the reverse MR analysis results due to weak instrumental bias, and we cannot completely dismiss the possibility of a reverse causal association. Our exploration was primarily at the genetic level, without investigating the significant influence the unique structure and metabolites of bacteria might have on KD’s emergence and progression. The gut microbiota’s complexity and diversity are prominent, yet our study didn’t analyze how KD might be influenced by the multifaceted interrelations among various microbiota types amidst gut microbiota instability.

## Conclusions

Our findings indicate that gut microbiota may not play a direct genetic causal role in KD. However, future studies utilizing larger datasets and more sophisticated MR methods are essential to conclusively address this question. However, this does not rule out the possibility of associations beyond genetic causation. Confounding factors may explain the associations observed in previous observational studies. To confirm these findings, future studies will require more advanced MR methodologies, larger GWAS datasets, and improved genetic instruments.

## Supplementary information

Supplementary Figures

Supplementary Table

## Data Availability

Publicly available datasets were analyzed in this study. These data can be found here: MiBioGen consortium (www.mibiogen.org, accessed on 5 May 2024) and GWAS Catalog consortium (https://www.ebi.ac.uk/gwas/studies/GCST90013537, accessed on 5 May 2022).
