# Supplementary material for: No causal association between gut microbiota and kawasaki disease: a two-sample mendelian randomization study
Source: Pediatr Res. 2025 Jan 25;98(2):423–30. doi: 10.1038/s41390-025-03878-5 (PMC12454152; doi:10.1038/s41390-025-03878-5)
Supplement: Supplementary file 1 — Supplementary Figures [file 41390_2025_3878_MOESM1_ESM.pdf]

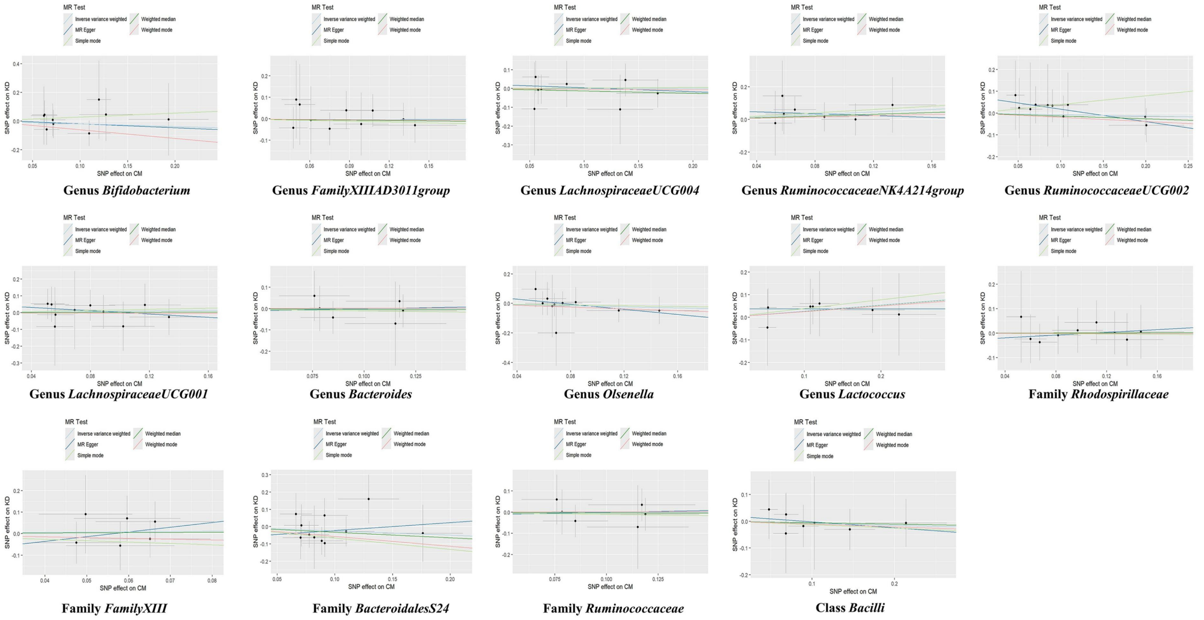

Supplementary Figures S1 Scatter plots for the causal association between gut microbiota and KD.

This collection of scatter plots illustrates the causal associations between various gut microbiota taxa and KD. Each plot represents a different genus or family of gut microbiota, with the x-axis showing the SNP effect on the gut microbiota and the y-axis showing the SNP effect on KD. Each black dot signifies a single SNP, with the line segment depicting the 95% CI. The slope of the straight line reflects the causal estimation derived from the MR method. In this visualization, the light blue line corresponds to the IVW method, the blue line represents the MR Egger method, the light green represents the Simple Mode, the pink represents the Weighted Mode, and the dark green line represents the Weighted Median method.

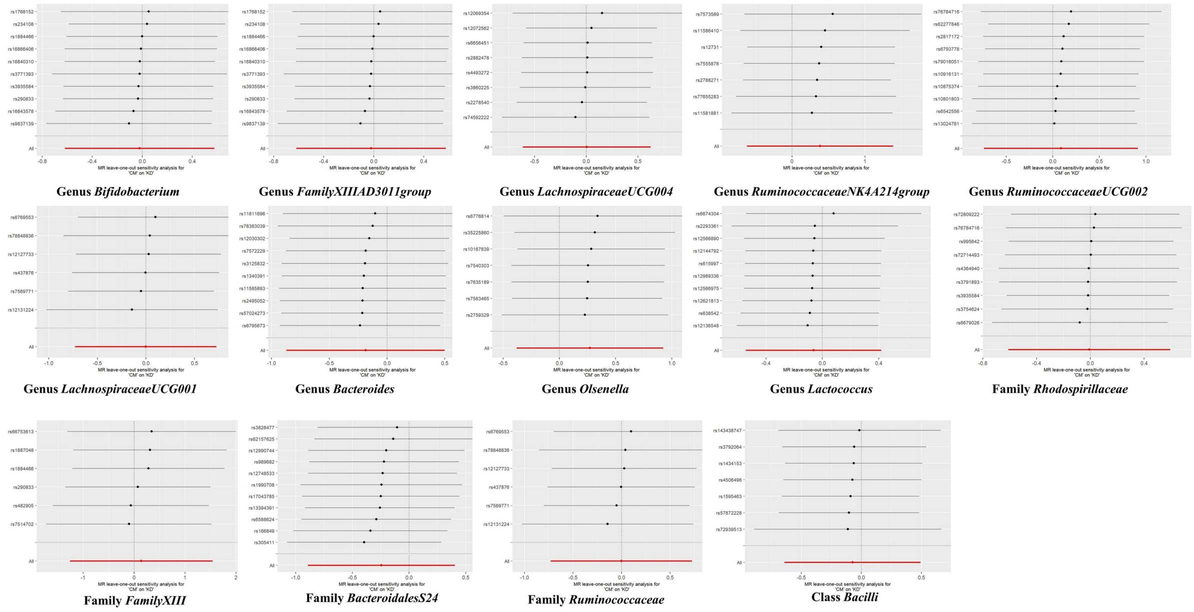

Supplementary Figures S2 Leave-one-out plot of the causal relationships between gut microbiota and KD.

This series of leave-one-out plots assesses the robustness of the causal relationships between specific gut microbiota taxa and KD. Each plot corresponds to a different taxonomic group, with the x-axis representing the effect of SNPs on KD when each SNP is excluded one at a time from the analysis, and the y-axis representing the cumulative effect of all other SNPs. The leave-one-out sensitivity analysis demonstrates that the causal relationships between the gut microbiota taxa and KD are robust and not unduly influenced by any single SNP.

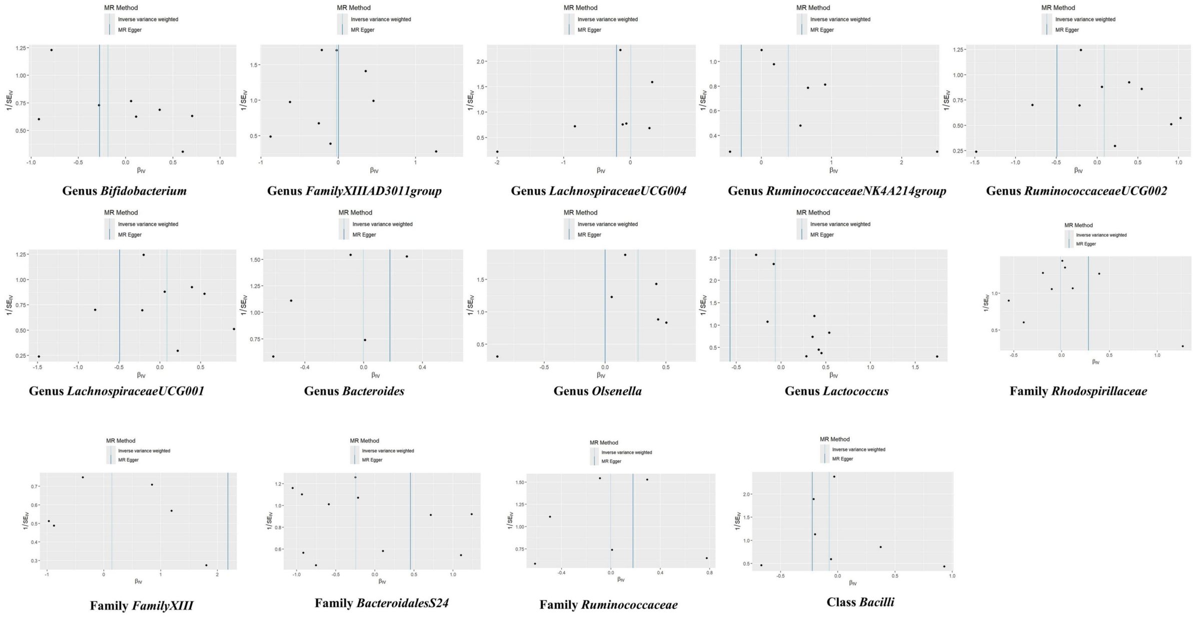

Supplementary Figures S3 Funnel plot of the causal relationships between gut microbiota and KD.

This collection of funnel plots evaluates the publication bias and the precision of the causal effect estimates for various gut microbiota taxa in relation to KD. Each plot corresponds to a different taxonomic group, with the x-axis representing the precision of the effect estimates (1/SE, where SE is the standard error) and the y-axis representing the effect size (log OR, where OR is the odds ratio). The funnel plots indicate that the causal relationships between the gut microbiota taxa and KD are generally free from publication bias and that the effect estimates are precise. The consistency of the grey dots around the blue line across different SNPs further supports the robustness of the findings. The light blue represents the IVW method, and the dark blue represents the MR Egger method.
